# Hydrogel capsules as new approach for increasing drying survival of plant biostimulant gram-negative consortium

**DOI:** 10.1007/s00253-023-12699-7

**Published:** 2023-08-22

**Authors:** Martha Chaparro-Rodríguez, German Estrada-Bonilla, Jaiver Rosas-Pérez, Martha Gómez-Álvarez, Mauricio Cruz-Barrera

**Affiliations:** 1 Bioproducts Department, Corporación Colombiana de Investigación Agropecuaria (AGROSAVIA), Km 14 Vía Bogotá a Mosquera, Mosquera, Colombia; 2 Departamento de Farmacia, Facultad de Ciencias, Universidad Nacional de Colombia, Bogotá, Colombia; 3 Agricultural Microbiology Laboratory, Tibaitatá Research Center, Corporación Colombiana de Investigación Agropecuaria (AGROSAVIA), Km 14 Vía Bogotá a Mosquera, Mosquera, Colombia

**Keywords:** Encapsulation, Desiccation tolerance, Formulation, Scaling-up, Biopolymer, Storage stability

## Abstract

**Abstract:**

Several plant growth–promoting bacteria (PGPB) are gram-negative, and their cell viability is affected during the bio-inoculant production. Hence, formulation-drying processes provide challenges that limit the adoption of these beneficial microorganisms in sustainable agricultural production. Among delivery system strategies for gram-negative PGPB, the encapsulating cells in biopolymeric materials are emerging as a promising alternative. This research aims to evaluate the effect of additives and crosslinking agents on the survival of the consortium of *Herbaspirillum frisingense* AP21, *Azospirillum brasilense* D7, and *Rhizobium leguminosarum* T88 in hydrogel capsules. Three crosslinkers and diverse potential drying protectors were tested. Calcium gluconate provides notable consortium survival advantages regarding colony-forming units (CFUs) (losses of up to 4 log CFU) compared to calcium lactate and calcium chloride (up to 6 log CFU). Additives such as skimmed milk, whey protein, and Gelita® EC improve the recovery of viable cells after the drying process, demonstrating an increase in cell survival of the three bacteria by up to 4 log CFU. The combination of these substances into a capsule prototype extends the storage stability of bacterial consortium up to 3 months at 18 ± 2 °C. This study expands the knowledge for formulating gram-negative PGPB consortium, regarding the crosslinker and drying protector relationship on encapsulation processes with drying survival and further storage stability performance.

**Key points:**

• *Hydrogel immobilization formulation approach for PGPB consortium*

• *Enhancing drying survival of gram-negative PGPB consortium*

• *Increasing storage stability of PGPB consortium at 18 °C*

## Introduction

Pastures and forages need the development and application of new alternatives and sustainable biotechnological solutions, focused on reducing the use of synthetic chemical fertilizers. Currently, the demand for plant biostimulants is increasing worldwide owing to the necessity of eco-friendly biotechnologies products to preserve the environment (O'Callaghan et al. 2022; Xu and Geelen 2018). Thereby, the global biostimulant market is projected to grow from $3.14 billion in 2022 to $4.14 billion by 2025 (Madende and Hayes 2020). The increase in the biostimulant market is driven because of the upsurge in demand for sustainable agriculture and reduced use of synthetic chemical fertilizers.

The plant growth–promoting bacteria (PGPB) are considered biostimulants with multifunctional attributes that help to reduce the application of chemical synthesis fertilizers. PGPB provides great advantages to plants through nitrogen fixation, the increase of soil phosphorus availability, the siderophore production (Lobo et al. 2019; Soares 2022), and secreting antibacterial and antifungal agents (Dudeja et al. 2021). The PGPB can induce the systemic resistance of the plant and the production of phytohormones, such as indole acetic acid and cytokinins, and improve the biological activities of the soil, increasing the absorption rate of nutrients by plants and their response to biotic and abiotic factors (Elnahal et al. 2022; Fadiji et al. 2022; Hakim et al. 2021). Although several studies claim that individual microbes can exert benign effects on plants, it is increasingly palpable that when a microbial consortium (more than two) interacting with microorganisms is related, additive or synergistic results can be expected (Santoyo et al. 2021).

Previous investigations showed the capacity of *Herbaspirillum frisingense* AP21, *Azospirillum brasilense* D7, and *Rhizobium leguminosarum* T88 to fix nitrogen, solubilize mineralize phosphorus, and mitigate the deleterious effect of drought (Santos-Torres et al. 2021). This PGPB consortium is an outstanding plant biostimulant, which is capable to solubilize and to mineralize phosphorus, reducing by up to 50% the application of nitrogen and phosphorus fertilization (Cortes-Patino et al. 2021; Pardo-Diaz et al. 2021).

An important issue in the use of these PGPBs in many cases is that the results obtained in laboratory or greenhouse conditions are not easily transferable to the field, especially when it comes to gram-negative bacteria. Hence, these non-spore-forming bacteria are more sensitive to deleterious factors, such as desiccation, changes in pH, and sudden modifications in temperature among others (Berninger et al. 2018a; Lobo et al. 2019; O'Callaghan et al. 2022). Owing to this challenge and to promote the use of this type of bacteria, it is crucial to design protective formulations. Gram-negative bacteria require adequate treatment, using formulations of protective devices to support their efficiency at the target site and aid practical use by farmers (Berninger et al. 2018b; Orozco-Mosqueda et al. 2021; Young et al. 2006). Historically, biofertilizers are distinguished by different types of formulations based on the use of vehicles such as peat, talc, activated carbon, granules, powders, in liquid formulations, and in cell immobilization by encapsulation (Herrmann and Lesueur 2013; Mahanty et al. 2017). During formulation processes, there are numerous parameters that can influence the survival of cells. The drying processes are generally the most critical and demanding to the development of solid formulations (Berninger et al. 2018b; Greffe and Michiels 2020).

To mitigate the drying sensitivity of gram-negative PGPB during formulation, possible solutions lie on selecting a suitable drying method, the pre-conditioning by osmotic or oxidative stress, the activation of exopolysaccharide secretion in the consortium formulation (strains that can produce exopolysaccharides and provide protection to bacteria that do not produce), the external application of drying protectants, and the use of encapsulated formulations among others (Berninger et al. 2018b; Cruz Barrera et al. 2020; Greffe and Michiels 2020; Perez et al. 2018). Among formulation alternatives, the inclusion of PGPB through encapsulation in a polymeric matrix is getting popularity (Mendoza-Labrador et al. 2021; Riseh et al. 2021; Szopa et al. 2022; Vejan et al. 2019). Within encapsulation, natural and synthetic polymers can be used, which facilitate the formation of hydrogels, retaining substantial among water, such as alginate, carrageenan, agarose, polyacrylamides, polystyrene, starch, carbohydrates, cellulose, chitosan, dextrans, lignin, and polyurethane (Cesari et al. 2020; Humbert et al. 2017; Schoebitz et al. 2013). The encapsulation in these materials protects strains from biotic and abiotic stress (pollutants, soil antagonists, temperature, dryness, ultraviolet light, stress mechanical) and provides them with a beneficial microenvironment. This leads to a prolonged shelf life and maintenance of its metabolic activity (Cruz Barrera et al. 2020; Przyklenk et al. 2017; Schoebitz et al. 2013). However, there are few reports considering gram-negative PGPB consortium (more than two strains) as an active ingredient in biopolymeric capsules and even less information regarding drying survival performance. Although it has been little researched, some crosslinking agents in the encapsulation process such as calcium gluconate may improve the cell survival of gram-negative bacteria (Humbert et al. 2017; Schoebitz et al. 2012). Hence, further efforts should be devoted to encourage encapsulation technologies in the PGPB consortium, particularly in the choice of polymers and additives (Balla et al. 2022).

Considering the challenges above, this research aims to increase the drying survival of gram-negative PGPB consortium (*H. frisingense* AP21, *A. brasilense* D7, and *R. leguminosarum* T88) by evaluating new excipients in a hydrogel capsule formulation, such as the effect of protective drying substances and crosslinking agents.

## Materials and methods

### Microorganisms

The PGPB consortium composed of *R. leguminosarum* strain T88 (SAMN15498640), *H. frisingense* strain AP21 (SAMN15498633), and *A. brasilense* strain D7 (SAMN16830199) was supplied by the Microorganisms Germplasm Collection of Microorganisms (Corporación Colombiana de Investigación Agropecuaria (AGROSAVIA), Mosquera, Colombia). AP21 and D7 were isolated from *Pennisetum clandestinum* (kikuyo) plants, and T88 was isolated from red glover (Cortes-Patino et al. 2021).

### Inoculum preparation

The strains D7 and AP21 were cultivated in a solid culture medium optimized for the growth of PGPB bacteria containing (g L^−1^) glutamate (28.33), yeast extract (2.92), HPO_4_3H_2_O (1.34), MgSO_4_ 7H_2_O (0.5), and FeCl_3_ (0.02) agar plates at 30 ± 0.5 °C for 24 h (Moreno-Galván et al. 2012). The strain T88 was cultivated on (g L^−1^) glutamate (13.67), yeast extract (0.73), H_2_PO_4_ (0.48), MgSO_4_7H_2_O (0.2), NaCl (0.1), and CaCl_2_ (0.1) at 30 ± 0.5 °C for 72 h. Subsequently, the Petri dishes of each bacterium (D7, T88, and AP21) were scraped, placed in saline solution (0.85% w/v), and centrifuged separately. Afterward, the pellet of each strain was resuspended in a 5% w/v gelatin and sucrose solution, and the cell viability (CFU) and the OD_600_ were assessed and adjusted prior to the encapsulation procedure.

### Reagents

Amidated pectin (Grinsted® LA 410, esterification degree 31%, amidation degree 19%) and calcium gluconate were provided by CIMPA (Bogotá, Colombia). All other materials were of analytical reagent grade and were used as received.

### Excipient selection by the compatibility test

A compatibility test was carried out to observe the effect of excipients (crosslinkers and drying protectors) on the cell viability of *R. leguminosarum* T88, *A. brasilense* D7, and *H. frisingense* AP21. Calcium chloride (5% p/v), calcium gluconate (5% w/v), and calcium lactate (5% w/v) as crosslinkers were evaluated. Within the polymeric matrix, the drying protectors (trehalose (5% w/v), guar gum (1% w/v), arabic gum (2% w/v), skim milk (2.5% p/v), whey protein (2.5% w/v), soy flour (2.5% w/v), cornstarch (1% w/v), gelatin (2% w/v), and Gelita® EC (Gelita AG, Eberbach, Germany; 2% w/v) were assessed. Bacterial suspensions of D7, T88, and AP21 were prepared in a sterile saline solution (0.1% p/v), and the concentration was adjusted at 0.3 OD_600_ (~ 10^7^ UFC mL^−1^). The compatibility test was performed using a 96-well microplate (Greiner Bio-One, Frickenhausen, Germany, Ref: 655,161), and 240 µL of the mixture was placed in each well between the excipient and the bacterial suspension at a 1:1 ratio (each bacterium was evaluated with each excipient). Subsequently, the plate reader was set at the following conditions: 25 °C, shaking (high) for 72 h (D7 and AP21), and 96 h (T88), reading the OD_600_ every hour. Afterward, the maximum specific speed (µ_max_ h^−1^) of each bacterium with each substance was determined. Besides, the cell viability in colony-forming units (CFU mL^−1^) was evaluated after 3 and 5 days of contact with the excipients for the strains D7, AP21, and T88 respectively. The assay controls were the saline solution (positive control) in contact with each bacterium, where the bacteria were expected to maintain their initial concentration or show minimal growth, and saline solution with chloramphenicol (1% w/v) (negative control), where it was expected to decrease the bacteria viability. Each treatment was evaluated with 3 replicates. An incompatible excipient (crosslinking agent or drying protector) was considered upon the bacteria viability reduction. Conversely, a compatible excipient did not decrease the bacteria growth, and its concentration was equal to or higher than the positive control.

### Hydrogel capsule preparation

The encapsulation of the bacteria consortium was carried out by means of the ionic gelation method (Cruz Barrera et al. 2020; Vemmer and Patel 2013). Briefly, polymer-matrix suspension followed by a suspension of the three bacteria was prepared. The polymer solution was prepared with amidated pectin. The pectin was dissolved in ultrapure water to a final concentration of 4.0% w/v, maintaining constant stirring at 600 rpm and 70 ± 5 °C using a stirring plate (Santos-Díaz et al. 2022). Then, the bacteria consortium suspension was added to the polymer matrix, a sample of the matrix was taken, and the cell concentration (CFU mL^−1^) of each bacterium was calculated. After 15 min of stirring, the suspension was transferred to a 20-mL syringe and dripped through a cannula (diameter 2.1 × 0.8 mm, Sterican, B. Braun Melsungen AG, Melsungen, Germany) into the stirred crosslinking agent solution. The ionic gelling reaction took place immediately, and the resulting hydrogel capsules were kept for 10 min in the stirred crosslinker. Furthermore, the capsules were separated from crosslinking agent solution using a sieve (mesh size: 1.0 mm). Then, the capsules were dried off by convection at 25 ± 2 °C and at 70 ± 5% of relative humidity. For each trial, the viability of three bacteria was determined before and after the drying process by ten capsules suspended in 10 mL of citric acid (0.03 M) and sodium carbonate (0.05 M) sterile solution (pH 7 ± 2) for 20 min on a rotary shaker at 1680 rpm and at 18 °C. After complete dissolution, serial dilutions were plated onto a solid culture medium optimized for each PGPB and incubated at 30 °C for 48 h. Afterward, the number of colonies was counted. The response variables were the encapsulation efficiency (%) and the cell survival (%) after the drying process.

The results were expressed as a percentage of survival on a logarithmic basis (log CFU) (Cruz Barrera et al. 2020). From the viability results (CFU capsule^−1^) at wet capsules (*N*_*O*_) and after dry capsules (*N*), the drying survival was calculated using

$$Cell \;survival = log \;No/ log \;N$$

### Crosslinker effect on bacteria consortium drying survival

Three crosslinking agents such as calcium chloride, calcium gluconate, and calcium lactate were evaluated. The solutions were prepared in ultrapure water at 0.1 M, calcium chloride (1.18% w/v), calcium gluconate (4.3% w/v), and calcium lactate (2.18% w/v) and were sterilized (121 °C, 15 psi, 20 min). The gluconate solution was allowed to warm to complete dissolution before sterilization. Cell survival was evaluated before and after drying.

### Excipient effect on drying survival

Selected excipients as drying protectors were screened. The additives tested in this experiment were trehalose, guar gum, arabic gum, skim milk, whey protein, soy flour, cornstarch, gelatin, and Gelita® EC. The substances were included at 1% w/w within the polymeric matrix, and subsequently, the bacterial cells of the three bacteria were uniformly incorporated using a stirring plate at 300 rpm for 10 min. Afterward, the mixture was dropped on the crosslinking agent, and their cell survival within the capsules before and after drying was assessed.

### Storage stability of the selected prototype

The formulation prototypes that include calcium gluconate as a crosslinking agent and the skim milk (P1), whey protein (P2), and Gelita® EC (P3) as a drying protector were selected. The prototypes were formulated under the previously mentioned conditions. The wet capsules were dried by convection at 25 ± 2 °C for 28 h.

The viability of bacterial strains T88, D7, and AP21 in the prototypes P1, P2, and P3 was determined up to 3 months of storage. Thus, samples of each prototype (1 g, 3 replicates per time, and per treatment) were packed in sealed aluminum bags and then stored under the conditions at 8 ± 2 °C, 18 ± 2 °C, and 28 ± 2 °C. Every month, three replicates of 10 capsules from one bag were added to 10 mL of citrate buffer (pH 7 ± 2) for 20 min on a rotary shaker at 1600 rpm and at 18 °C. After complete disintegration dissolution, serial dilutions were plated onto a solid culture medium optimized for the growth of PGPB and incubated at 30 °C for 96 h. Finally, the number of colonies was quantified. The viability results (CFU capsule^−1^) were at time zero (t_0_) and after storage (t_15 days_, t_30 days_, t_60 days_, t_90 days_).

### Scanning electron microscopy (SEM)

The surface morphology of the capsules was analyzed by scanning electron microscopy (SEM). Briefly, the selected capsule prototype sample was fixed by immersion in 2% glutaraldehyde for 10 min and then washed with ethanol gradients (50%, 80%, and 99% v/v) for 15 min each. At each step, the samples were dried in an extraction cabinet to remove the solvent. Afterward, the samples were placed in a desiccator with silica gel for 48 h as an additional dehydration step. Cross-sections were cut off from the capsules, which were then subjected to the same fixation and drying process prior to the SEM observation. The fixed and dried samples were coated with a 30-nm gold microfilm using a metallizer (Q150R ES, Quorum Technologies, United Kingdom) at sputter current 60 mA, 40 s, and tooling factor 2.30. Observations were made using a Quanta 200 microscope with Everhart–Thornley SE and solid-state BSE detectors (SEMTech Solutions Inc., North Billerica, MA, USA).

### Statistical analysis

Data were analyzed using SPSS Statistics v.2 software (SPSS, Chicago, IL). Data were checked for normality and homogeneity of variances using the Shapiro–Wilk and Bartlett tests, respectively (α = 0.05). The significant effects of the treatments on the measured variables were determined using a one-way analysis of variance (ANOVA) followed by Tukey’s post hoc test for comparison of the means of treatments. The level of significance was settled at *p* < 0.05. For the stability study, a completely randomized design was carried out with a factorial arrangement adjusted for repeated measures over time. The determination of significant effects was carried out with the restricted maximum likelihood method (REML), and for the separation of means, the Tukey multiple comparison method was used. These procedures were performed with the PROC GLIMMIX procedure from the SAS Enterprise (Cary, NC, USA) guide 8.3 program.

## Results

### Excipient selection by the compatibility test

**Fig. 1:**
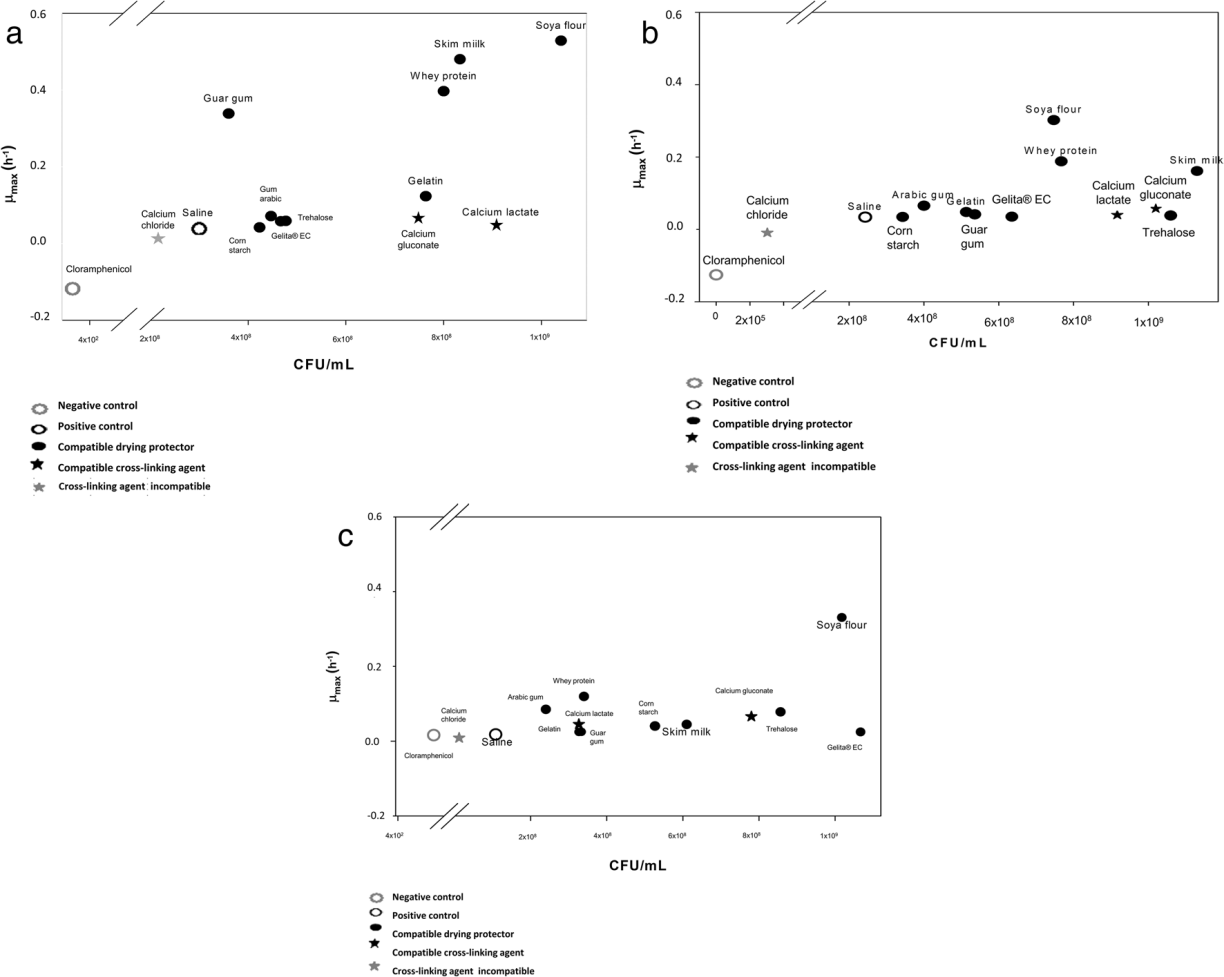
Compatibility results of different crosslinking agents and drying protectants with bacteria. Specific speed (µ_max_ h.^−1^) of each bacterium vs. colony-forming units (CFU/mL) at the time of highest concentration with each substance was determined. The cell viability was evaluated after 3 and 5 days of contact of the excipients for the strains D7, AP21, and T88 strain respectively. **a** *A. brasilense* strain D7. **b** *H. frisingense* strain AP21. **c** *R. leguminosarum* strain T88. CFU/mL Tukey (*p* < 0.05), µ_max_ Kruskal–Wallis (*p* < 0.05)

The compatibility of *R. leguminosarum* T88, *A. brasilense* D7, and *H. frisingense* AP21 with various excipients was expressed in terms of the maximum specific growth rate (µ_max_ h^−1^), estimated at the time point in which higher cell concentration for each substance was found. In Fig. 1a, A*. brasilense* D7 in contact with most substances such as calcium lactate, calcium gluconate, skim milk, trehalose, guar gum, acacia gum, gelatin, cornstarch, Gelita® EC, whey protein, and soy flour showed a final cell concentration and also a µ_max_ higher than the control (saline solution), which was 2.97 × 10^8^ CFU mL^−1^ and 0.040 h^−1^. Interestingly, calcium chloride reached a lower final concentration than the control of 2.23 × 10^8^ CFU mL^−1^ and a µ_max_ < 0.0062 h^−1^.

Despite the fact that the viability and µ_max_ of the bacteria obtained with calcium chloride were lower than the control, there were no significant differences compared to the positive control (saline solution). Thus, for *A. brasilense* strain D7, all the substances were compatible with the bacterium (*F*_13.41_ = 2.09; *p* = 0.0493). The substances with superior cell concentration and µ_max_ were obtained with soybean meal (1.04 × 10^9^ CFU mL^−1^ and 0.5283 h^−1^), skim milk (8.33 × 10^8^ CFU mL^−1^ and 0.4802 h^−1^), and serum protein (8.0 × 10^8^ CFU mL^−1^ and 0.4802 h^−1^), indicating a possible greater compatibility of *A. brasilense* D7 with these substances. Similar results were obtained with the bacteria *H. frisingense* AP21 and *R. leguminosarum* T88 (Fig. 1b, c), and with most of the substances evaluated, the bacteria presented final concentrations higher than those of the control. These two bacteria presented µ_max_ greater than those of the control at 0.0337 h^−1^ for the *H. frisingense* AP21 bacteria and 0.0176 h^−1^ for *R. leguminosarum* T88. Indicating that the bacteria present compatibility with most of the crosslinking agents and drying protectors evaluated. However, the AP21 and T88 bacteria in contact with the calcium chloride crosslinking agent demonstrated an incompatibility, obtaining a concentration and µ_max_ significantly lower than control, being for *H. frisingense* AP21 3.03 × 10^5^ CFU mL^−1^ and 0.0092 h^−1^ and for *R. leguminosarum* T88 of 1.24 × 10^7^ CFU mL^−1^ and 0.0009 h^−1^ respectively (*F*_13.41_ = 4111; *p* < 0.05). The substances with the highest cell concentration and the highest µ_max_ for the *H. frisingense* AP21 bacteria were skim milk (1.13 × 10^9^ CFU mL^−1^, 0.1613 h^−1^), soy flour (7.47 × 10^8^ CFU mL^−1^, 0.3018 h^−1^), and serum protein (7.67 × 10^8^ CFU mL^−1^, 0.1880 h^−1^). For *R. leguminosarum* T88 bacteria, the soy flour (1.0 × 10^9^ CFU mL^−1^, 0.3306 h^−1^), Gelita® EC (1.07 × 10^9^ CFU mL^−1^, 0.0247 h^−1^), trehalose (8.57 × 10^8^ CFU mL^−1^, 0.0782 h^−1^), and calcium gluconate (7.8 × 10^8^ CFU mL^−1^, 0.0654 h^−1^) promoted relative high cell concentration.

### Crosslinker effect on bacteria consortium drying survival

**Fig. 2:**
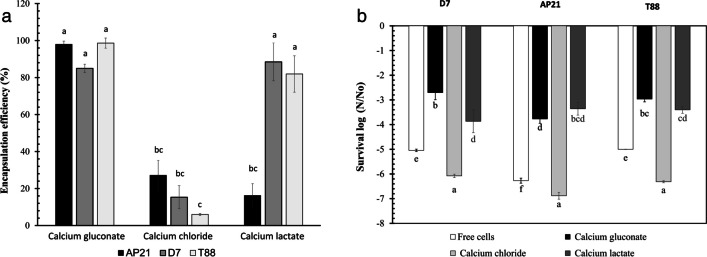
Evaluation of three crosslinking agents. **a** Encapsulation efficiency of the three bacteria using three crosslinking agents (calcium gluconate, calcium lactate, calcium chloride). Different letters represent significant differences according to Tukey’s multiple comparison test at *p* ≤ 0.05. **b** Survival percentage of bacteria *R. leguminosarum* T88, *A. brasilense* D7, and *H. frisingense* AP21 in beads using three crosslinking agents (calcium gluconate, calcium lactate, calcium chloride) after subjecting the capsules to a convection drying process in a drying room at 25 ± 2 °C. Different letters represent significant differences according to Tukey’s multiple comparison test at *p* ≤ 0.05

The encapsulation of the three bacteria was carried out by means of the ionic gelation method, evaluating three crosslinking agents: calcium chloride, calcium gluconate, and calcium lactate at 0.1 M. The dried capsules reached a moisture content of < 6% and a_w_ < 0.4. The crosslinking agents significantly affected the encapsulation efficiency of the three bacteria with the polymer matrix used (*F*_8.26_ = 118, *p* < 0.05). With calcium chloride, encapsulation efficiencies were lower than 50%, being significantly lower than those obtained with calcium gluconate, which reached encapsulation efficiencies > 80% for the three bacteria (Fig. 2a). Regarding the calcium lactate crosslinking agent, there were no significant differences with calcium gluconate for T88 and D7 bacteria, but significant differences were presented for AP21. Interestingly, for the cell survival of the three bacteria after the drying process, a significant effect of the crosslinking agent is observed (*F*_11.35_ = 1340, *p* < 0.05). For the variable cell survival (log *N*/*No*), which is presented in Fig. 2b, a lower value of log CFU indicates a greater protection to the drying process, suggesting a lower loss of viability of the bacteria. Calcium chloride as a crosslinker of polymeric matrix produced a greater cell loss compared to calcium gluconate and calcium lactate, with a reduction of more than 6 log CFU for the three bacteria (Fig. 2b). Conversely, calcium gluconate provided a significantly higher cell survival than calcium lactate for D7 bacteria. Calcium gluconate performed the best encapsulation efficiency for the three bacteria and the highest cell survival results for bacteria D7 and T88, and this crosslinking agent was selected for further drying protector analysis.

### Excipient effect on drying survival

**Fig. 3:**
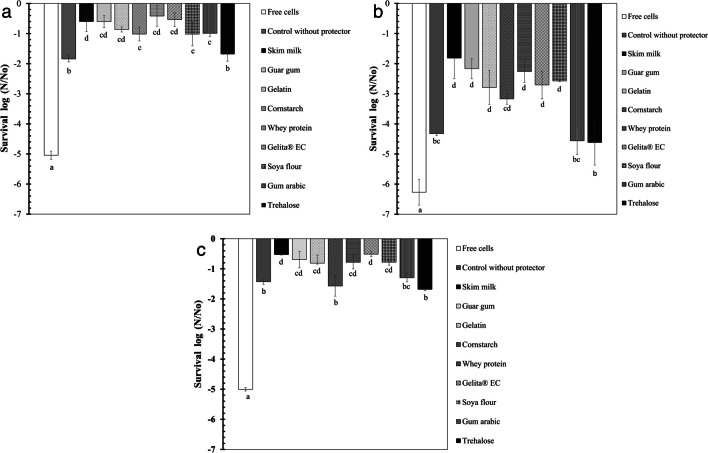
Survival percentage of bacteria after subjecting the beads to a convection drying process in a drying room at 25 ± 2 °C including different drying protectors in the polymeric matrix. **a** *A. brasilense* D7. **b** *H. frisingense* AP21. c R. leguminosarum T88. Different letters represent significant differences according to Tukey’s multiple comparison test at *p* ≤ 0.05

To improve the cell survival of the three bacteria, different drying protectants were evaluated (skim milk, trehalose, guar gum, gum arabic, gelatin, corn starch Gelita® EC, whey protein, and soybean meal). The materials were incorporated into the polymeric matrix at 1% w/w. The capsule preparation was carried out by ionic gelation using calcium gluconate (0.1 M) as a crosslinker. The results of the effect of the drying protectants on the cell survival of the bacterium *A. brasilense* D7 are presented in Fig. 3a**.** Comparing the unencapsulated cells of *A. brasilense* D7 with the encapsulated cells, using calcium gluconate without adding any drying protector (control) shows a significant increase in the cell survival of the bacteria (*F*_11.35_ = 120, *p* < 0.05). When the control is compared to the treatments that include drying protectors, a significant increase in cell survival was evidenced with the protectants: skim milk, guar gum, gelatin, cornstarch, Gelita® EC, whey protein, flour soy, and arabic gum. However, with the drying protectants, a greater protection of this bacterium is obtained; thus, whey protein reached a loss of cell viability of 0.41 log CFU, followed by skim milk with 0.59 log, Gelita® EC with 0.53 log CFU, and guar gum with 0.60 log CFU. The results of the effect of the drying protectants on the cell survival of *H. frisingense* AP21 are presented in Fig. 3b. There was a significant higher cell survival in AP21 than in the control (*F*_11.35_ = 34.4: *p* ≤ 0.05), losing 4.32 l log CFU compared to the free cells with 6.27 log CFU.

The formulation prototypes that include skim milk, guar gum, gelatin, Gelita® EC, whey protein, and soybean meal protectants present a significant less loss of viability with respect to the control. The excipients that presented the highest cell survival for the *H. frisingense* AP21 strain were skim milk, guar gum, whey protein, and Gelita® EC, with a log CFU loss of 1.81, 2.16, 2.26, and 2.70, respectively. The results of the effect of the drying protectants on the cell survival of the bacterium *R. leguminosarum* T88 are presented in Fig. 3c. A lower cell survival of the unencapsulated free cells of *R. leguminosarum* T88 is presented compared to the control. The cell loss of the unencapsulated cells was significantly greater than 5 log CFU compared to the encapsulated cells without applying any protector (*F*_11.35_ = 84.5, *p* ≤ 0.05), which was 1.42 log CFU. The prototypes that include skim milk, guar gum, gelatin, Gelita® EC, whey protein, and soy flour drying protectants presented a statistically significant protection of viability with respect to the control (without drying protectors). The protectants that presented the highest cell survival for *R. leguminosarum* T88 bacteria were skim milk, guar gum, Gelita® EC, and whey protein with a loss of 0.52, 0.69, 0.51, and 0.78 log CFU, respectively.

### Storage stability of the selected prototype

**Fig. 4:**
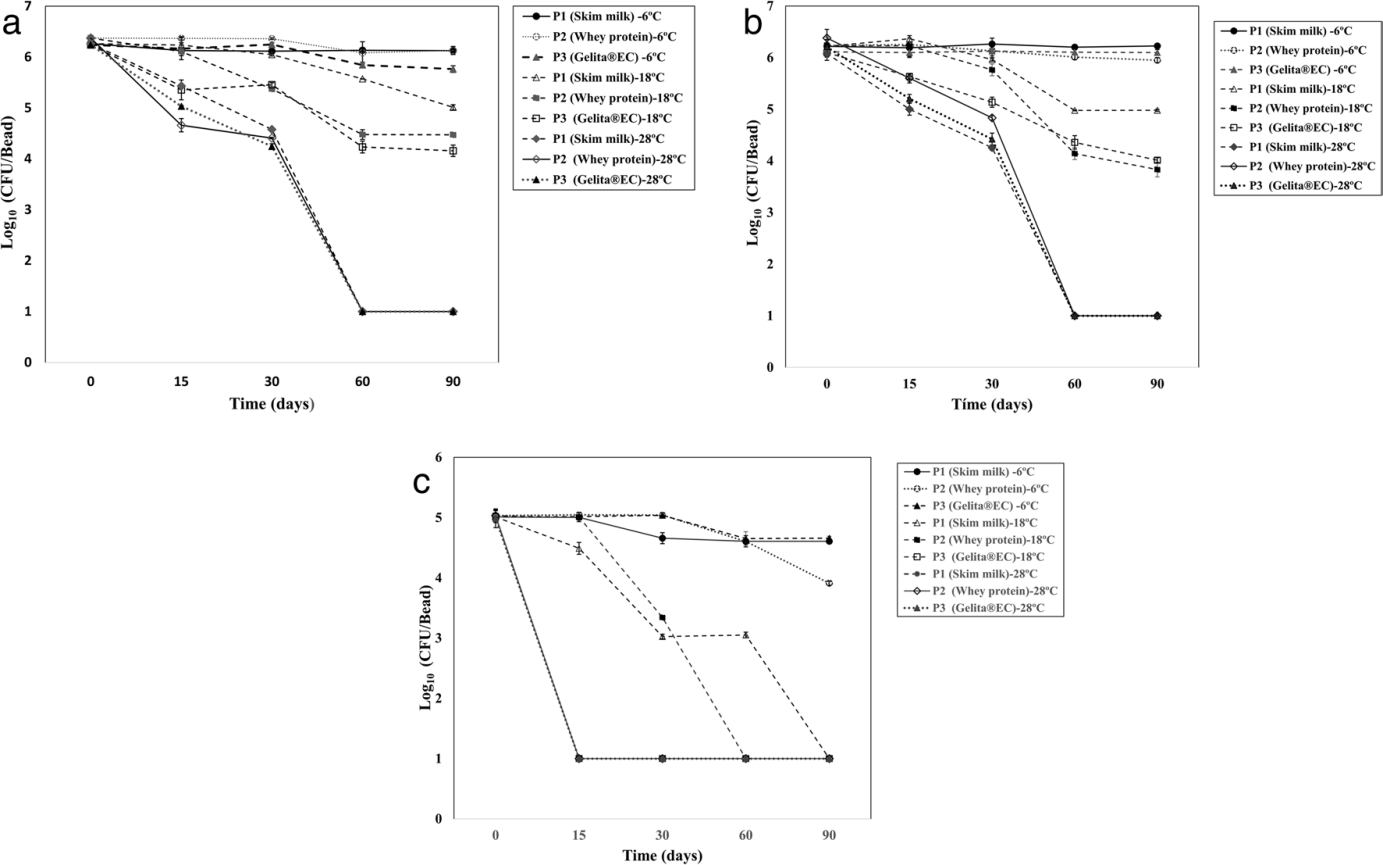
Viability of bacteria (log CFU/bead) within prototypes stored at 6 ± 2 °C, 18 ± 2 °C, and 28 ± 2 °C during 90 days. **a** *A. brasilense* D7. **b** *R. leguminosarum* T88. **c** *H. frisingense* AP21

To carry out the preliminary stability study, three prototypes were assessed. These prototypes were formulated with skim milk, whey protein, and Gelita® EC at 1%. For the three prototypes, calcium gluconate was used as a crosslinking agent based on the previous assay. The results of the stability over time of each bacterial strain D7, T88, and AP21 with the prototypes evaluated and at the three temperatures are presented in Fig. 4a, b, c, respectively. The criterion to determine the greater stability of the evaluated prototypes was the viability over time. The temperature at which the prototypes and the bacteria were significantly more stable was the temperature of 6 ± 2 °C, followed by 18 ± 2 °C (*F*_2270_ = 27,797; *p* ≤ 0.0001). The D7 bacterium showed the highest stability, being significantly more viable compared to the AP21 bacterium that was the most affected (*F*_2270_ = 18,503; *p* ≤ 0.0001) (Fig. 4a). When the prototype was analyzed as the main factor, the prototype that showed a significantly higher viability with respect to the other prototypes was skim milk, followed by whey protein and Gelita® EC (*F*_2405_ = 479.04; *p* ≤ 0.0001).

**Fig. 5:**
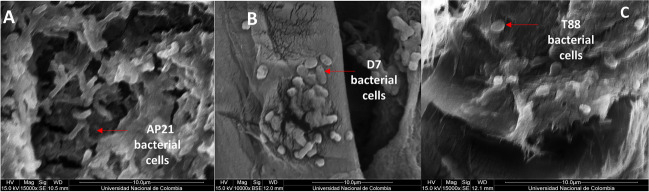

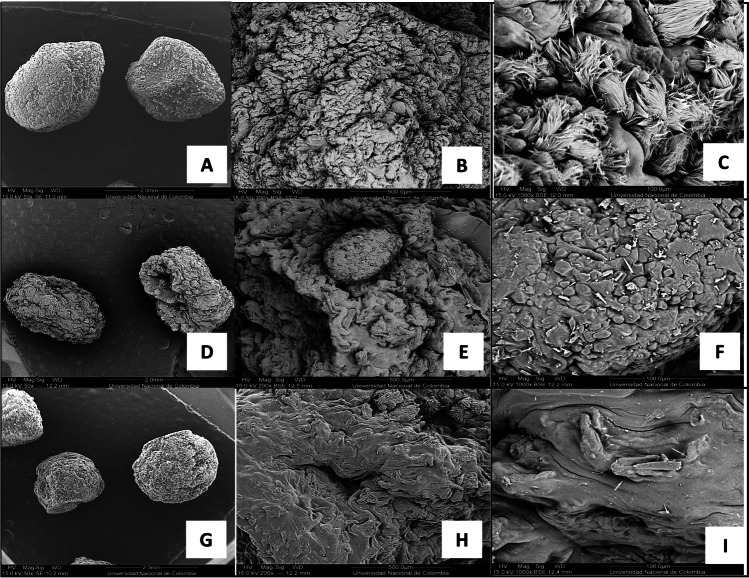
**a** Micrographs obtained by scanning electron microscopy (SEM) of the bacteria. (**A**) *H. frisingense* AP21 at × 15,000. (**B**) *A. brasilense* D7 at × 10,000. (**C**) *R. leguminosarum* T88 at × 15,000. **b** Micrographs obtained by scanning electron microscopy (SEM) of the capsule prototypes using the crosslinking agents. (**A**)–(**C**) Calcium chloride (× 50, × 200, × 1000). (**D**)–(**F**) Calcium lactate (× 50, × 200, × 1000). (**G**)–(**I**) Calcium gluconate (× 50, × 200, × 1000)

To verify the immobilization of the cells of the consortium, SEM studies and the topographies of the capsules were carried out depending on the variation of the crosslinking agent. Figure 5a shows the morphology of free-cell bacteria by SEM. In Fig. 5b, it is important to point out the smooth surface of the capsule surface provided by calcium gluconate compared to the rough and cracking surface by calcium chloride.

## Discussion

The encapsulation of microorganisms in polymers involves a technique that can considerably improve their survival against noxious surrounding factors, such as chemicals, pH variation, and deleterious agents generated during the formulation process, such as drying (Humbert et al. 2017; Przyklenk et al. 2017). In the encapsulation process, the components that are incorporated into any formulations are important; here, the excipients that are part of the capsules were compatible with the microorganisms immobilized, avoiding toxic effects on cells over time (Cortés-Rojas et al. 2021; Deaker et al. 2012).

In the study, the compatibility among crosslinking agents and the drying protectors and the bacteria *R. leguminosarum* T88, *A. brasilense* D7, and *H. frisingense* AP21 was evaluated*.* The three bacteria were compatible with the drying protectors assessed and with the crosslinking calcium gluconate and calcium lactate except for calcium chloride at the tested concentrations. The concentration tested for the three crosslinkers was 5% w/v (Cruz Barrera et al. 2020; Schoebitz et al. 2013, 2012). However, for calcium chloride, it is a high concentration compared to other studies, where they use ~ 1% and 3% w/v (Mendoza-Labrador et al. 2021). This excessive concentration of calcium chloride may promote osmotic stress in cells that cause the mortality of the three bacteria and therefore their incompatibility.

The excipients such as skim milk, soy flour, whey protein, trehalose, and Gelita® EC showed a faster growth with higher µ_max_ values up to 0.528 µ_max_ h^−1^ with the three bacteria. These substances are widely used in different microorganism formulations, and some have also been evaluated as drying protectors (Cortés-Rojas et al. 2021; Rodriguez-Salazar et al. 2009; Schoebitz et al. 2012). The bacteria growth with the different substances demonstrates their capacity to metabolize these substances and to proliferate. Thus, some of these substances can serve as carbon sources and are easily consumed by several bacteria (Arguelles 2000).

The three bacteria obtained a higher percentage of encapsulation using calcium gluconate as a crosslinking agent followed by calcium lactate and calcium chloride; this may be because crosslinking agents can crosslink pectin through (i) electrostatic attractive force between cations (Ca^2+^, Zn^2+^), (ii) covalent amide bond (between the activated –COOH functional group of the crosslinker with the e-NH_2_ group of pectin), (iii) covalent imine bond (between the –CHO functional group of the crosslinker with the e-NH_2_ group of the pectin), (iv) structure of the H bond (between the polyphenolic group –OH with a different type of amino acid of the pectin molecule), (v) formation of coordinate bonds, and (vi) the Maillard reaction (proteins, carbonyl group of a pectin residue) reducing the sugar of pectin, which reacts with the e-NH_2_ group of the protein (Lara-Espinoza et al. 2018; Mitra et al. 2011). With all these bonding patterns, pectin-based biopolymeric materials are stabilized. In this case, calcium gluconate has carboxyl groups that allow it to react with the e-NH_2_ group of the pectin and also has a greater amount of OH with respect to chloride and lactate, which will allow it to form a greater number of H bonds with the pectin, generating greater crosslinking and therefore greater encapsulation efficiency for bacteria (Cruz Barrera et al. 2020).

Contrarily, the calcium chloride crosslinking agent showed the lowest encapsulation efficiency for the three bacteria, being < 50% for all of them. This may be due in part to the negative effect that this substance presented when the compatibility tests were carried out, and possibly, this contact in the process could decrease the bacterial viability of the three strains. Similarly, encapsulation efficiencies of 40 to 61% were obtained by other researchers, who used calcium chloride (0.1 M) as a crosslinking agent to encapsulate *A. brasilense* (Joe et al. 2014; Zago et al. 2019). Additionally, the crosslinking agent calcium chloride may generate gels with greater porosity, with holes and irregular topography, as observed in the SEM micrographs (Fig. 5b).

An increase in the degree of crosslinking can reduce the free volume within of the hydrogel network structure, thereby reducing the size of the pores. Hence, in polymers such as pectin, electrostatic attraction forces can occur between cations (Ca^+2^, Zn^+2^), covalent amide bonds, covalent imine bonds, H^+^ bonds, and formation of coordinate bonds. The degree of pectin methoxylation can also influence its lower degree which leads to a more compact gel formation (Lara-Espinoza et al. 2018). Here, pectin had a methyl esterification degree of 31% and an amidation degree of 19%, being low and allowing to form more compact gels.

Calcium gluconate showed a protective effect on *A. brasilense* D7 and *R. leguminosarum* T88 after a convective drying process of bacterial encapsulation. Calcium gluconate is classified as osmoprotective, although it is not well known how it performs the protective effect on the membranes of microbial cells (Schoebitz et al. 2012). Upon water elimination during the drying process, the gluconate changes to a dissociated form of cyclic ester, and this structure presents a strong structural resemblance to cyclic monosaccharides. Thus, sugars have the ability to form amorphous glasses in the dry state and thus can slow down diffusion processes due to the high viscosity in the glassy state and protect the membrane (Humbert et al. 2017). The drying protection effect of calcium gluconate on bacteria, even fungi, has also been evidenced by other authors (Cruz Barrera et al. 2020; Humbert et al. 2017; Schoebitz et al. 2012).

The drying protectors that protected the three bacteria were skim milk, whey protein, Gelita® EC, and guar gum. Skim milk is made up of lactose and milk protein, compounds that can interact with the cell membrane and help to maintain its integrity in a similar way to non-reducing disaccharides like sucrose (Malafronte et al. 2015). These substances such as lactose can form hydrogen bonds between the hydroxyl groups and the phosphate of the lipid bilayer of the cell membrane (Hildebrand et al. 2008). Hydrogen bonds allow lactose to act as a replacement for water molecules and thus maintain membrane stability (Hildebrand et al. 2008). Additionally, milk and whey protein can form a protective coating on the bacterial cell wall when they interact with calcium. The protective effect of skim milk in drying processes on bacteria of the same genera evaluated has been evidenced in *Azospirillum lipoferum* (Fages 1990) and on *A. brasilense* (Bashan et al. 2002). Gelita® EC is made up of pectin and gelatin; thus, the protective effect of gelatin and pectin on other microorganisms has been evidenced by other authors, who evaluated different protectors for spray drying of the bacterium *Bifidobacterium bifidum* BB-12 and *Trichoderma koningiopsis* and found the greatest protection with gelatin, pectin, and arabic gum (Cortés-Rojas et al. 2021; Salar-Behzadi et al. 2013).

The results obtained in this study demonstrated a minimal loss of viability of the PGPB consortium in the three formulation prototypes stored at 6 ± 2 °C for 3 months. After 3 months of storage, *A. brasilense* D7 strain viability in prototypes P1 and P2 maintains the same logarithm, at a concentration of 10^6^ CFU/g or 10^8^ CFU/g capsules. Similarly, when two rhizobacteria such as *A. brasilense* and *Raoultella terrigena* were encapsulated in a matrix of starch with alginate, the number of viable cells remained constant at around 10^9^ CFU/g of dry capsules during 1 year of storage at 4 °C (Schoebitz et al. 2012). To highlight, herein is the first report considering *H. frisingense* within a bacteria consortium and formulated as dried hydrogel capsules.

The behavior at 18 ± 2 °C allowed us to determine that all the prototypes presented a loss of viability after 90 days of storage. However, prototype P1 protects the consortium, especially D7 and T88, with a viability reduction of 1.22 and 1.51 log CFU respectively, remaining at a concentration of 10^5^–10^7^ CFU g^−1^ of dried capsule. Regarding the storage at 28 °C, the greatest loss of viability was presented for the three bacteria at 90 days of storage (> 5 log units). This could be due to the fact that high storage temperatures cause a greater activity of the bacteria and consumption of energy and nutrients; if the bacteria lose their energy and nutrients, they cannot maintain normal metabolism and gradually decrease the population (Żur et al. 2016). When an evaluation was made on the formulation of the consortium, no registered product was found at temperatures above 28 °C or 30 °C, and there are very few investigations in which formulations at this temperature have maintained their viability. An example is the encapsulation of *Rhizobium* sp. in dry alginate beads that after 8 months of storage started from 9.92 log CFU and reached 7.35 log CFU (Lobo et al. 2019; Thirumal et al. 2017).

To conclude, the drying protectants and the crosslinkers calcium gluconate and calcium lactate were compatible with the three bacteria at 0.1 M. The endophytic *H. frisingense* AP21 was the most sensitive bacterium to the convective drying process. Calcium gluconate as a crosslinker provided a high encapsulation efficiency > 80% and protection of the consortium gram-negative bacteria in the drying processes, serving as a drying protector. The skimmed milk, Gelita® EC, and whey protein drying protectants increased the desiccation tolerance of the three bacteria and reduced cell death up to 4 log CFU. The P1 prototypes maintained a stable bacterial viability at 18 ± 2 °C for 90 days. This research expands the knowledge on the PGPB consortium formulation techniques to promote the development of hydrogel beads as the delivery system.

## Data Availability

All datasets generated for this study can be provided upon request.
