# Correction: Hydrogel capsules as new approach for increasing drying survival of plant biostimulant gram‑negative consortium

**DOI:** 10.1007/s00253-023-12801-z

**Published:** 2023-09-27

**Authors:** Martha Chaparro‑Rodríguez, German Estrada‑Bonilla, Jaiver Rosas-Pérez, Martha Gómez‑Álvarez, Mauricio Cruz‑Barrera

**Affiliations:** 1 Bioproducts Department, Corporación Colombiana de Investigación Agropecuaria (AGROSAVIA), Km 14 Vía Bogotá a Mosquera, Mosquera, Colombia; 2 Departamento de Farmacia, Facultad de Ciencias, Universidad Nacional de Colombia, Bogotá, Colombia; 3 Agricultural Microbiology Laboratory, Tibaitatá Research Center, Corporación Colombiana de Investigación Agropecuaria (AGROSAVIA), Km 14 Vía Bogotá a Mosquera, Mosquera, Colombia

**Correction to: Applied Microbiology and Biotechnology**

**https://doi.org/10.1007/s00253-023-12699-7**

In the published version of the original paper, the name of the third author has been misspelled.

It should be corrected from “Javier Rosas-Pérez” to “**Jaiver Rosas-Pérez**”.

The original article has been corrected.

